# Circulating tissue inhibitor of metalloproteinases 1 (TIMP-1) at COVID-19 onset predicts severity status

**DOI:** 10.3389/fmed.2022.1034288

**Published:** 2022-11-29

**Authors:** Stefano Brusa, Daniela Terracciano, Dario Bruzzese, Mariano Fiorenza, Lucia Stanziola, Biagio Pinchera, Valeria Valente, Ivan Gentile, Antonio Cittadini, Ilaria Mormile, Mauro Mormile, Giuseppe Portella

**Affiliations:** ^1^Department of Translational Medical Science, University of Naples Federico II, Naples, Italy; ^2^Department of Public Health, University of Naples Federico II, Naples, Italy; ^3^Department of Clinical Medicine and Surgery, University of Naples Federico II, Naples, Italy

**Keywords:** COVID-19, fibrosis, TIMP-1, collagen metabolites, extrcellular matrix remodelling biomarkers

## Abstract

**Background:**

Systemic biomarkers for severity of SARS-CoV-2 infection are of great interest. In this study, we evaluated a set of collagen metabolites and extracellular matrix remodeling biomarkers including procollagen type III amino terminal propeptide (PIIINP), tissue inhibitor of metalloproteinases 1 (TIMP-1) and hyaluronic acid (HA) as prognostic indicators in COVID-19 patients.

**Methods:**

Ninety COVID-19 patients with the absence of chronic liver diseases were enrolled. Serum PIIINP, TIMP-1, and HA were measured and correlated with inflammatory indices and clinical variables. Patients were stratified for disease severity according to WHO criteria in two groups, based on the requirement of oxygen support.

**Results:**

Serum TIMP-1, but not PIIINP and HA was significantly higher in patients with WHO score ≥5 compared to patients with WHO score <5 [PIIINP: 7.2 (5.4–9.5) vs. 7.1 (4.5–9.9), *p* = 0.782; TIMP-1: 298.1 (20.5–460) vs. 222.2 (28.5–452.8), *p* = 0.01; HA: 117.1 (55.4–193.7) vs. 75.1 (36.9–141.8), *p* = 0.258]. TIMP-1 showed moderate correlation with CRP (r = 0.312, *p* = 0.003) and with LDH (r = 0.263, *p* = 0.009). CRP and serum LDH levels were significantly higher in COVID-19 patients with WHO score ≥5 compared to the group of patients with WHO score < 5 [15.8 (9–44.5) vs. 9.3 (3.4–33.8), *p* = 0.039 and 373 (282–465) vs. 289 (218–383), *p* = 0.013, respectively].

**Conclusion:**

In patients with COVID-19, circulating TIMP-1 was associated with disease severity and with systemic inflammatory index, suggesting that TIMP-1 could represent a promising non-invasive prognostic biomarker in COVID-19 patients. Interestingly, our results prompted that serum TIMP-1 level may potentially be used to select the patients for therapeutic approaches targeting matrix metalloproteases pathway.

## Introduction

The Severe Acute Respiratory Syndrome Coronavirus 2 (SARS-CoV-2) is the infective agent responsible for Coronavirus Disease 2019 (COVID-19). SARS-CoV-2 stimulates the immune system leading to cytokine storm (1) with markedly increased levels of several cytokines as IL–1α, IL-1β, IL-6, and TNF-α (2). In addition, an increase of neutrophils count and decreased count of lymphocytes have been observed (3). COVID-19 infection also leads to ROS generation (4) and coagulation cascade favoring the risk of thrombosis (5). Some subjects infected by SARS-CoV-2 developed a broad range of pathologies including not only pneumonia, acute respiratory distress syndrome (ARDS), respiratory failure but also systemic inflammation and multiorgan failure (1). Severe COVID-19 was associated with massive alveolar damage with loss of lung architecture, leading to ventilatory failure. A recently published article (6) reported two types of lung fibrosis after COVID-19. The first with a diffuse fibrotic alveolar damage is characterized by extracellular matrix deposition resulting in fibrosis; these patients require intubation, mechanical ventilation and/or extracorporeal membrane oxygenation (ECMO). The second is the post-COVID pulmonary fibrosis, diagnosed by the combination of clinical, radiological, and pathological information. In about 25% of patients with severe COVID-19 disease (WHO Severity Grade 3 and 4), a restrictive ventilatory defect was revealed. Thus, there is a compelling clinical need to identify circulating fibrosis markers in COVID-19. Ideally, these markers should be non-invasive, able to mirror the extent of fibrosis and to reflect disease progression and therapeutic response. SARS coronavirus induced up-regulation of Type I collagen, leading to pulmonary pro-fibrotic responses (7). Thus, collagen metabolism plays a key role in COVID-19 clinical picture. Several blood parameters have been evaluated as predictors of COVID-19 severity. However, at present, still no validated biomarkers are reliably used in routine clinical practice.

Procollagen type III amino terminal propeptide is the peptide released during the biosynthesis and depositing of type III collagen (8). TIMP-1 is an inhibitor specific for extracellular matrix (ECM) degradation enzymes (9). HA is a glycosaminoglycan engaged in the formation of ECM (10).

Elevated serum levels of PIIINP, HA or TIMP-1 were found to be increased in other diseases, such as in patients with systemic sclerosis (SSc) (11). High levels of PIIINP and HA were demonstrated to be unfavorable predictors for survival in SSc suggesting that these markers could be useful to predict other fibrotic lesions (12).

In this study we investigated the potential role of PIIINP, HA and TIMP-1 as prognostic markers in COVID-19 patients.

## Materials and methods

### Patients

We enrolled 90 adult hospitalized patients with a diagnosis of SARS-CoV-2 infection, confirmed by molecular analysis (RT-PCR) of the nasopharyngeal swab (13).

Patients were stratified for COVID-19 disease severity based on WHO scale (14). According to this classification patients were classified as: (1), asymptomatic, not hospitalized (2), symptomatic, not hospitalized, independent; (3), symptomatic, not hospitalized, assistance needed; (4), hospitalized, not requiring supplemental oxygen; (5), hospitalized, requiring oxygen by non-invasive mechanical ventilation (mask or nasal prongs); (6–9), hospitalized, requiring high-flow oxygenation and/or invasive mechanical ventilation; and 10, death.

Our study population was divided according to the severity of COVID-19 at the time of sampling into the following groups: (1) hospitalized COVID-19-positive patients requiring no respiratory support or oxygen support only (WHO ≤5); (2) hospitalized COVID-19-positive patients requiring invasive or non-invasive mechanical ventilation (WHO > 5).

The study was conducted in compliance with the Declaration of Helsinki. The protocol was approved by the Ethical Committee of the University Federico II of Naples (prot. no. 140/20). Informed consent was obtained from all individuals. At the time of sampling, laboratory parameters, clinical and demographic data were recorded.

### Biomarkers

Fasting blood samples were obtained. Sera were frozen and stored at −80^°^C until measurements. Samples were assayed in an automated analyzer that performs magnetic separation enzyme immunoassay tests (ADVIA Centaur; Siemens Healthcare Diagnostics, Tarrytown, NY, United States) for Hyaluronic acid (HA), amino-terminal propeptide of type-III-procollagen (PIIINP) and tissue inhibitor of metalloproteinase type-1 (TIMP-1).

### Statistical analysis

All statistical analyses were performed using the R platform version 4.1.2. Standard descriptive statistics were used to describe the cohort: mean ± standard deviation (range) or median (25th; 75th percentile) (range) in case of numerical variables and absolute frequency with percentages for categorical factors. Accordingly, between-group comparisons were assessed using the *t*-test for independent samples, the Mann-Whitney U-test and the Chi-square test (or the Fisher exact test when appropriate). Median regression with bootstrapped standard errors was used to adjust the analysis for potential confounding factors.

## Results

A total of 90 COVID-19 patients (43 female and 47 male) were enrolled and classified for disease severity based on World Health Organization (WHO) stage. 68 (75.6%) COVID-19 patients with a WHO score <5 and 22 (24.4%) with a WHO score >5. Demographic and clinical features are showed in Table 1. Mean age was 58.6 ± 15.4 (range: 38–62) years, patients with WHO score >5 were significantly older than patients with WHO score <5 (*p* = 0.013); no differences in comorbidities at baseline were observed between the two groups. Median disease duration (time length to negativization) was 23 days (range 5–72 days) days with a longer disease duration in patients with a WHO score >5. In the overall cohort, 37 (41.6%) patients had a time length of negativization <21 days and 52 (58.4%) >21 days.

**TABLE 1:** Clinical and demographical characteristics of the study cohort stratified according to the World Health Organization (WHO) score at baseline.

	Overall (*n* = 90)	WHO score ≤5 (*n* = 68; 75.6%)	WHO score >5 (*n* = 22; 24.4%)	*P*-value
Age (years)	58.6 + −15.4 (20.6–93.9)	57.3 + −16.1 (20.6–86.9)	65.8 + −12.6 (39.3–93.9)	**0,013**
COPD	8 (11.9)	5 (10)	3 (17.6)	0,684
Diabetes	17 (25.4)	12 (24)	5 (29.4)	0,749
Hypertension	17 (25.4)	12 (24)	5 (29.4)	0,749
Arhythmias	3 (4.5)	2 (4)	1 (5.9)	1
Time to negativization; days	23 (19; 33) (5–72)	22 (18; 31) (5–72)	30 (21; 41) (9–51)	**0,045**
HRCT score				0,176
≤10	54 (60)	44 (64.7)	10 (45.5)	−
>10	36 (40)	24 (35.3)	12 (54.5)	−

Data are expressed as mean ± standard deviation (range); median (25th; 75th percentile) (range) or absolute frequency (percentage). COPD, chronic obstructive pulmonary disease; HRCT, High Resolution Computed Tomography. Bold values indicate the strong correlation.

Patients were classified also for High-Resolution Computed Tomography (HRCT) score, resulting in 54 subjects (60%) with a score <10 and 36 (40%) >10. Of note, lymphocyte number was significantly lower in patients with HRCT score >10 [670 (270–2540) vs. 880 (260–3350); *p* = 0.026).

**FIGURE 1:**
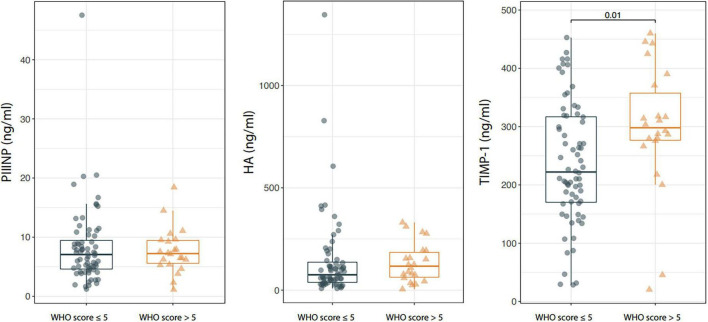
Box-plot showing serum procollagen type III amino terminal propeptide (PIIINP), Hyaluronic acid (HA), and tissue inhibitor of metalloproteinases 1 (TIMP-1) levels in patients with Coronavirus Disease 2019 (COVID-19) stratified according to the WHO score at baseline. Boxes are defined by Q1, Median (bold line) and Q3. Whiskers reach the minimum and the maximum of the distribution except for the presence of outliers, defined as data points below Q1–1.5*IQR or above Q3 + 1.5*IQR. To avoid overlapping a small amount of horizontal jitter was added. Q1, First quartile; Q3, Third quartile; IQR = Q3–Q1.

Serum TIMP-1 levels were significantly higher in patients with WHO score >5 than in those with a WHO score ≤5 [TIMP-1: 222.2 (20.5–460) vs. 298.1 (28.5–452.8), *p* = 0.010] and the difference was confirmed after adjusting the analysis for the age of patients through median regression (*p* = 0.003). On the contrary, no statistically significant difference was observed in serum PIIINP and HA between patients with severe and mild disease [PIIINP: 7.2 (1.1–18.4) vs. 7.1 (1.2–47.5), *p* = 0.782; HA: 117.1(4.7–331.1) vs. 75.1 (8.3–1345.9), *p* = 0.258; Figure 1].

As shown in Table 2, LDH and CRP values were significantly higher in patients with severe disease [LDH: 373 (193–670) vs. 289 (111–741), *p* = 0.013; CRP: 15.8 (1.3–222.5) vs. 9.3 (0.3–132.6), *p* = 0.039]. Table 3 showed that serum PIIINP, HA and TIMP-1 positively correlated with LDH levels (PIIINP: r = 0.264, *p* = 0.009, HA: r = 0.267, *p* = 0.008; TIMP-1: r = 0.263, *p* = 0.009).

**TABLE 2:** Distribution of blood parameters in patients stratified according to their World Health Organization (WHO) score at baseline.

	WHO score ≤5 (*n* = 68; 75.6%)	WHO score >5 (*n* = 22; 24.4%)	*P*-value
Hb g/dL	12.7 + −2.1 (7.7–18.1)	12.6 + −2.5 (7.6–16.1)	0,839
WBC (cells/μl)	6,565 (4,660; 9212.5) (2,380–17,090)	7,765(5682.5; 10147.5) (4210–21,150)	0,131
Neutrophils (N/mmc)	5,525 (3,545; 7,735) (1,000–13,600)	6,200 (4,385; 8007.5) (3,680–19,200)	0,064
Lymphocytes (N/mmc)	845 (602.5; 1267.5) (260–2,970)	635 (465; 1037.5) (270–3,350)	0,069
Platelets (cell/μL)	225,000 (185,500; 282,000) (55,000–363,000)	234,000 (134,750; 295,250) (31,000–607,000)	0,974
Fibrinogen (mg/dl)	581.7 + −173.2 (283–1,000)	563.2 + −187.2 (260–1,000)	0,685
INR	1.1 (1.04; 1.22) (0.8–3.84)	1.1 (1.03; 1.19) (0.8–1.33)	0,622
D-Dimer (mg/l)	1.02 (0.56; 1.69) (0.04–18.99)	0.94 (0.43; 2.38) (0.18–25.56)	0,794
Albumin (g/dl)	3.5 + −0.5 (2.6–4.5)	3.6 + −0.5 (2.5–5)	0,562
Total bilirubin mg/dl	0.65 (0.45; 0.85) (0.21–5.09)	0.74 (0.5; 1.06) (0.26–1.54)	0,289
Direct bilirubin mg/dl	0.29 + −0.13 (0.1–0.76)	0.34 + −0.17 (0.1–0.69)	0,255
Ferritin (ng/ml)	533.5 (267.8; 744.5) (40–2,000)	488 (149; 820) (39–2,000)	0,816
AST U/L	25 (20; 33.5) (9–141)	29 (23.5; 35.5) (8–176)	0,135
ALT U/L	27 (19; 42) (7–177)	35 (21.5; 55) (9–474)	0,318
LDH U/L	289 (218; 383) (111–741)	373 (281.8; 465) (193–670)	**0,013**
hsCRP mg/L	9.3 (3.4; 33.8) (0.3–132.6)	15.8 (9; 44.5) (1.3–222.5)	**0,039**
IL-6 (pg/ml)	13.2 (7.2; 28.5) (2.3–63.4)	19.6 (6.3; 59.9) (3.5–258)	0,276

Data are expressed as mean ± standard deviation (range); median (25th; 75th percentile) (range) or absolute frequency (percentage). Hb, haemoglobin; WBC, white blood cells; INR, international normalized ratio; AST, aspartate transaminase; ALT, alanine transaminase; LDH, Lactate dehydrogenase; hsCRP, high sensitive C-reactive protein; IL-6, interleukin-6. Bold values indicate the strong correlation.

**TABLE 3:** Correlation among procollagen type III amino terminal propeptide (PIIINP), Hyaluronic acid (HA), and tissue inhibitor of metalloproteinases 1 (TIMP-1) with inflammatory markers.

	HA	PIIINP	TIMP-1
Fibrinogen	−0.162 (0.124)	−0.05 (0.638)	0.151 (0.152)
hsCRP	0.147 (0.175)	0.162 (0.134)	**0.312 (0.003)**
Ferritin	−0.028 (0.816)	0.084 (0.48)	−0.053 (0.657)
IL-6	−0.003 (0.986)	0.008 (0.959)	0.082 (0.61)
albumin	−**0.387 (<0.001)**	−**0.362 (<0.001)**	−0.177 (0.079)
LDH	**0.267 (0.008)**	**0.264 (0.009)**	**0.263 (0.009)**

Bold values indicate the strong correlation.

Serum PIIINP and HA negatively correlated with albumin values (PIIINP: r = −0.362, *p* < 0.001; HA: r = −0.387, *p* < 0.001); circulating TIMP-1 levels positively correlated with CRP values (r = 0.312, *p* = 0.003).

## Discussion

Our study highlighted the significant positive correlation between changes of TIMP-1 and disease severity based on WHO classification, suggesting that TIMP-1 could serve as a non-invasive biomarker for prognosis in COVID-19.

Metzemaekers et al. (15) reported significantly higher levels of plasmatic tissue inhibitor of metalloproteinase 1 (TIMP-1) and of TIMP-1/MMP-9 complexes and significantly lower circulating total MMP activity in COVID-19 patients at intensive care unit (ICU) admission.

Our data showed that serum TIMP-1 in SARS-CoV-2 infected patients correlates with the WHO score and the CRP values, but not with HRCT score and time length of negativization.

These findings may reflect peculiar aspect of the involvement of TIMP-1 in the fibrotic process: TIMP-1 represent decreased collagen degradation and was a strong predictor of early fibrosis (16).

Considering the short disease duration and moderate disease severity of most of our study population, our data indicated that TIMP-1 could be a useful marker of fibrotic burden and disease prognosis in patients with COVID-19 at initial diagnosis. Several molecular mechanisms involving matrix metalloproteases pathway have been identified as relevant players in the clinical picture of COVID-19 (17). It has been recently shown that matrix metalloproteinase-9 (MMP-9) gene expression is increased in subjects infected with SARS-CoV-2 (18) and circulating MMP-9 levels were significantly associated with the risk of respiratory insufficiency (19) and with severity in COVID-19 patients (20). In fact, some authors previously demonstrated that metalloproteinases (MMPs) seem to play a key role in lung disease (21, 22). Severe COVID-19 shared many characteristics with sepsis (23) and plasma MMP-9 and tissue inhibitor of matrix metalloproteinase-1 (TIMP-1) have been also proposed as septic biomarkers (24, 25).

Other authors showed that periodontitis and diabetes have been associated with COVID-19 poor outcomes and both these diseases have been correlated with elevated MMP-8 levels (26, 27), further highlighting the role of MMPs as key players in COVID-19 risk and escalation.

Tissue damage during SARS-CoV-2 lung infection is associated with activation of members of the MMPs family (28, 29). Targeting MMPs pathway has been proposed as therapeutic strategy to counterbalance the host marked pro-inflammatory response to the SARS-CoV-2 infection (30). In addition of being MMP-inhibitor, TIMP-1 is independently proinflammatory and pro-growth-factor (31–33). Thus, the measurement of circulating TIMP-1 levels could be useful to assess the prognosis and to adopt a personalized treatment approach.

Serum PIIINP, TIMP-1, and HA are combined to calculate the Enhanced Liver Fibrosis (ELF) score, initially developed from a chronic liver disease cohort (34–36). Thus, it was expected that the algorithm was not readily applicable to COVID-19. Nevertheless, our results suggest the need to derive a COVID-specific algorithm based on the clinical performance of single analytes markers in SARS-CoV-2 infected subjects.

It should also be considered that serum collagen metabolites may be affected by age, diet and disease duration (16, 37). Thus, to prevent results misinterpretation, they could be better used for within-individual changes during follow-up.

This study has several limitations. First, the study population is small, second, data on the correlation of TIMP-1 levels and specific treatment are lacking, third, serial measurements to assess longitudinal modifications of serum collagen markers according to fibrotic changes are not available. Thus, larger samples are needed to obtain a better evaluation of TIMP-1 levels circulating levels as a prognostic biomarker in COVID-19 patients and to investigate its potential role in monitoring therapeutic response in different treatment subgroups of COVID-19 patients.

In conclusion, our study shed new light on the potential clinical utility of serum collagen metabolites and extracellular matrix remodeling as suitable markers of disease severity in COVID-19 patients. We unveil that changes in serum TIMP-1 significantly correlate with changes in clinical outcome. Collagen metabolites and extracellular matrix remodeling markers are worthy of further studies to assess their potential as prognostic and predictive biomarkers in COVID-19 patients. The identification of a COVID-specific index reflecting the fibrotic process in SARS-CoV-2 patients is strongly encouraged for its potential as a disruptive tool for clinical management.

## Data availability statement

The data that support the findings of this study are available from the corresponding author DT, (daniela.terracciano@unina.it), upon reasonable request and with permission of AOU Federico II.

## Ethics statement

The studies involving human participants were reviewed and approved by the Ethical Committee of the University Federico II of Naples (prot. no. 140/20). The patients/participants provided their written informed consent to participate in this study.

## Author contributions

DT and GP: conceptualization and writing—review and editing. SB, BP, LS, MF, VV, and IM: data curation. DB: formal analysis. GP: funding acquisition, methodology, supervision, and validation. SB, DT, MF, and LS: investigation. IG, GP, AC, and MM: resources. DT, IG, AC, MM, and GP: visualization. DT: writing—original draft. All authors have read and agreed to the published version of the manuscript.
